# Supplementary material for: (L)-Monomethyl Tyrosine (Mmt): New Synthetic Strategy via Bulky ‘Forced-Traceless’ Regioselective Pd-Catalyzed C(sp2)–H Activation
Source: Pharmaceuticals (Basel). 2023 Nov 10;16(11):1592. doi: 10.3390/ph16111592 (PMC10675785; doi:10.3390/ph16111592)
Supplement: Supplementary file 1 [file pharmaceuticals-16-01592-s001.zip › pharmaceuticals-2671535-supplementary.pdf]

Table of Contents

1. ^{1}H and ^{13}C NMR spectra ……………………………………………………………………………S1
2. Application on SPPS……………………………………………………………………………….S10

1. ^{1}H and ^{13}C NMR spectra

Compound 2:

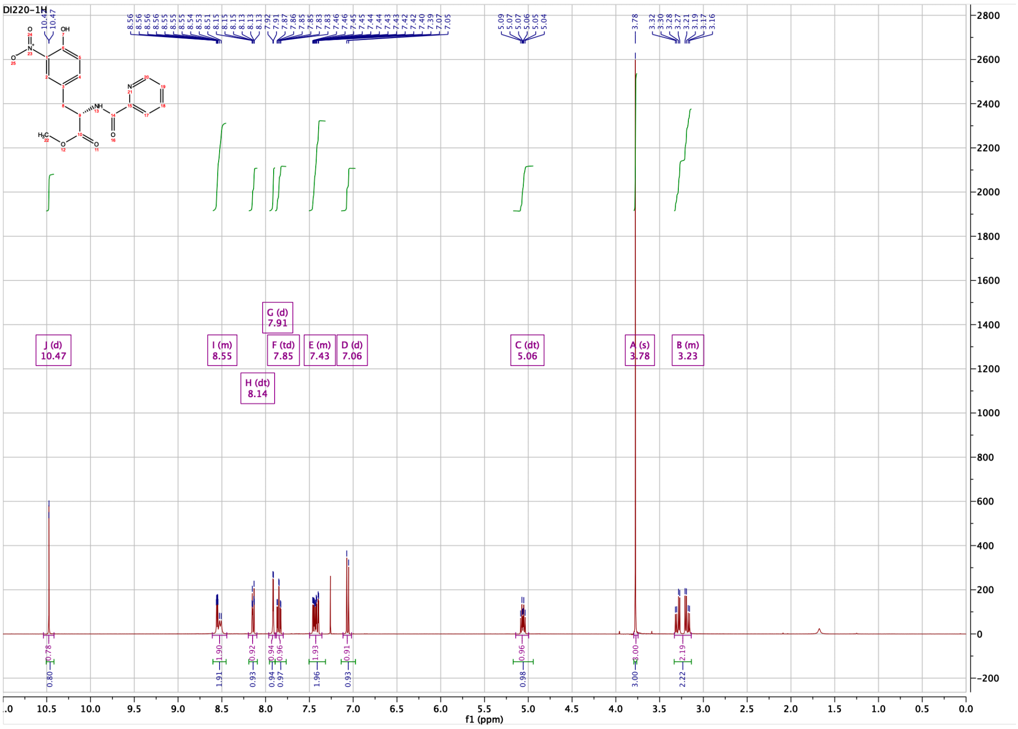

^{1}H NMR (400MHz, $CDCl_3$) compound **2**

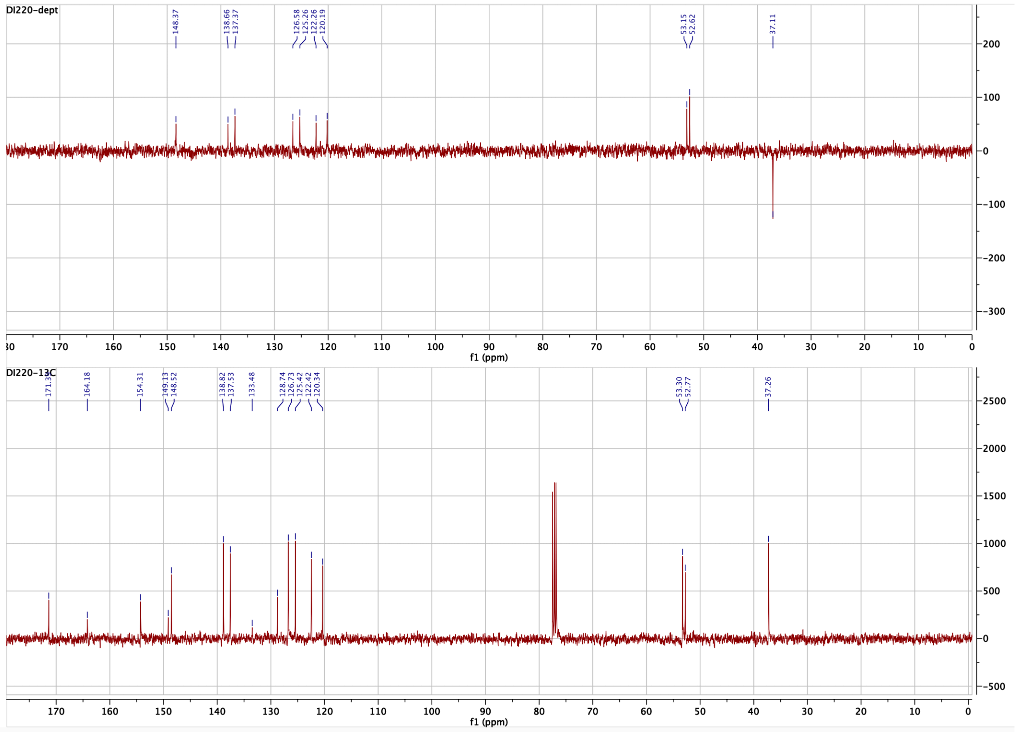

^{13}C and DEPT NMR (101 MHz, $CDCl_3$) compound **2**

Compound 3:

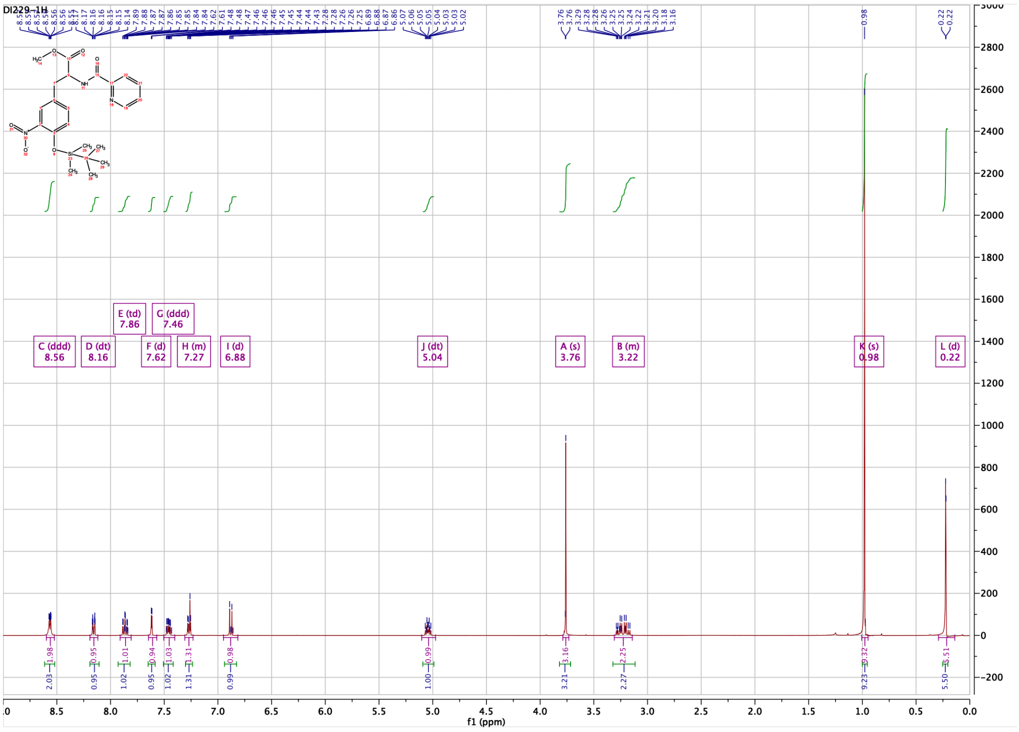

^{1}H NMR (400MHz, $CDCl_3$) compound **3**

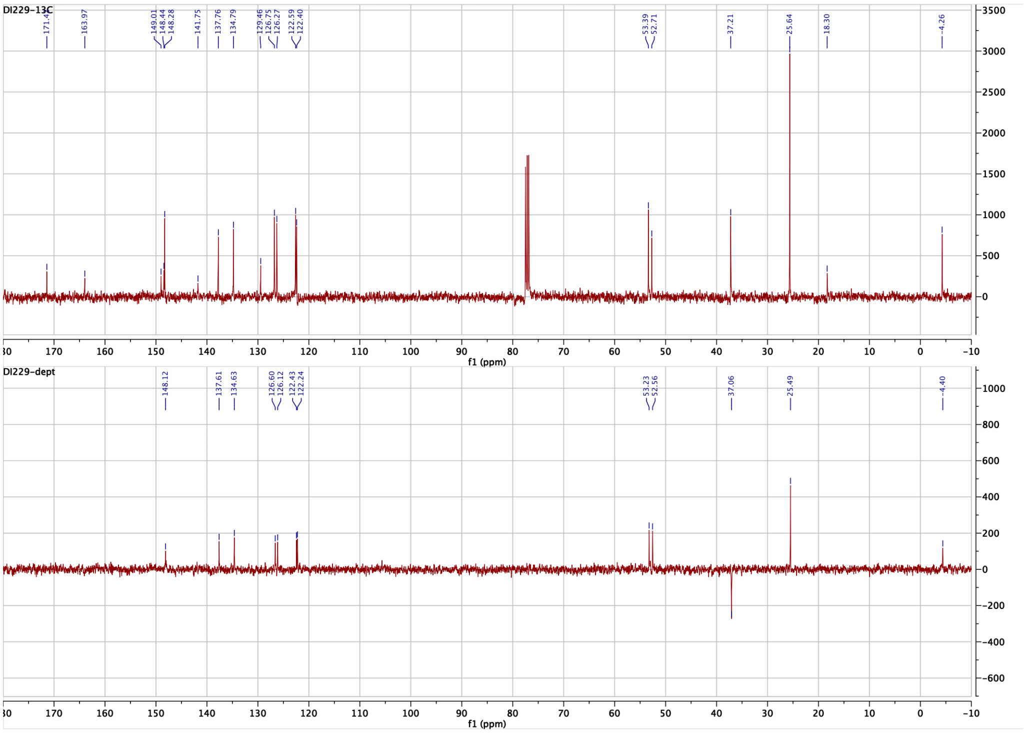

^{13}C and DEPT NMR (101 MHz, $CDCl_3$) compound **3**

Compound 4:

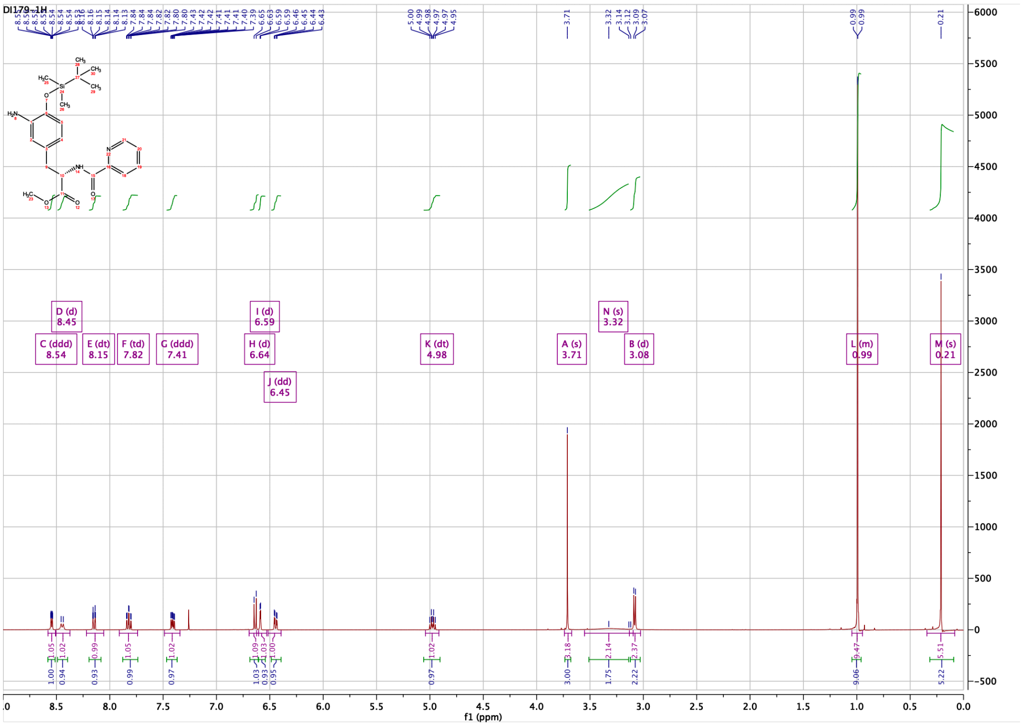

^{1}H NMR (400MHz, $CDCl_3$) compound **4**

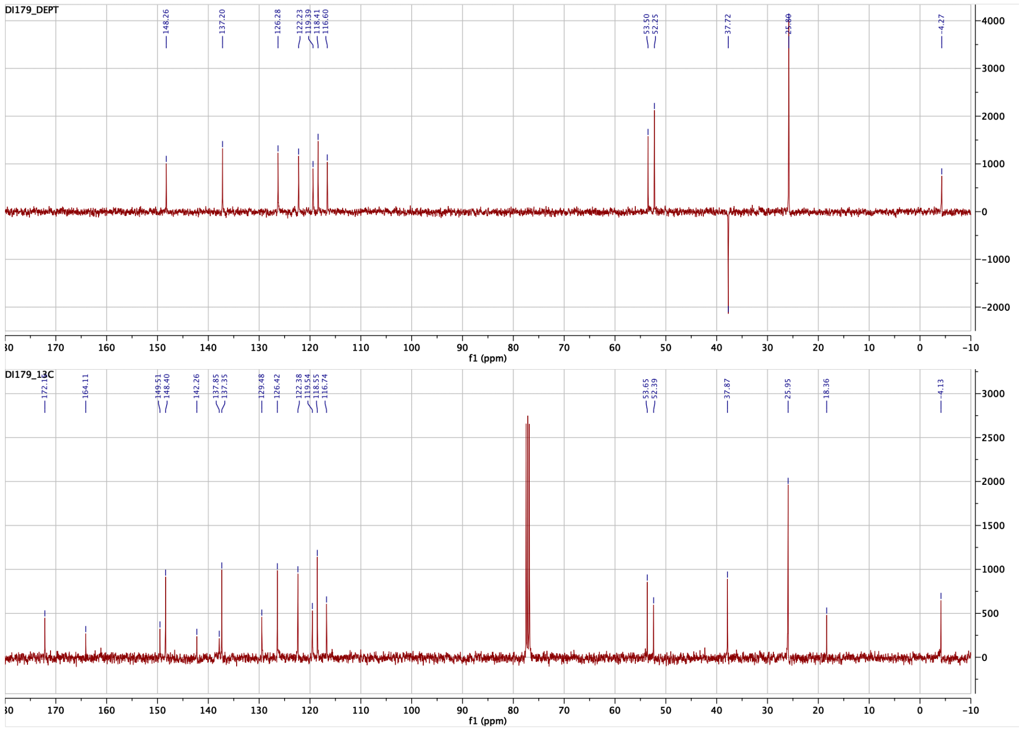

^{13}C and DEPT NMR (101 MHz, $CDCl_3$) compound **4**

Compound 5:

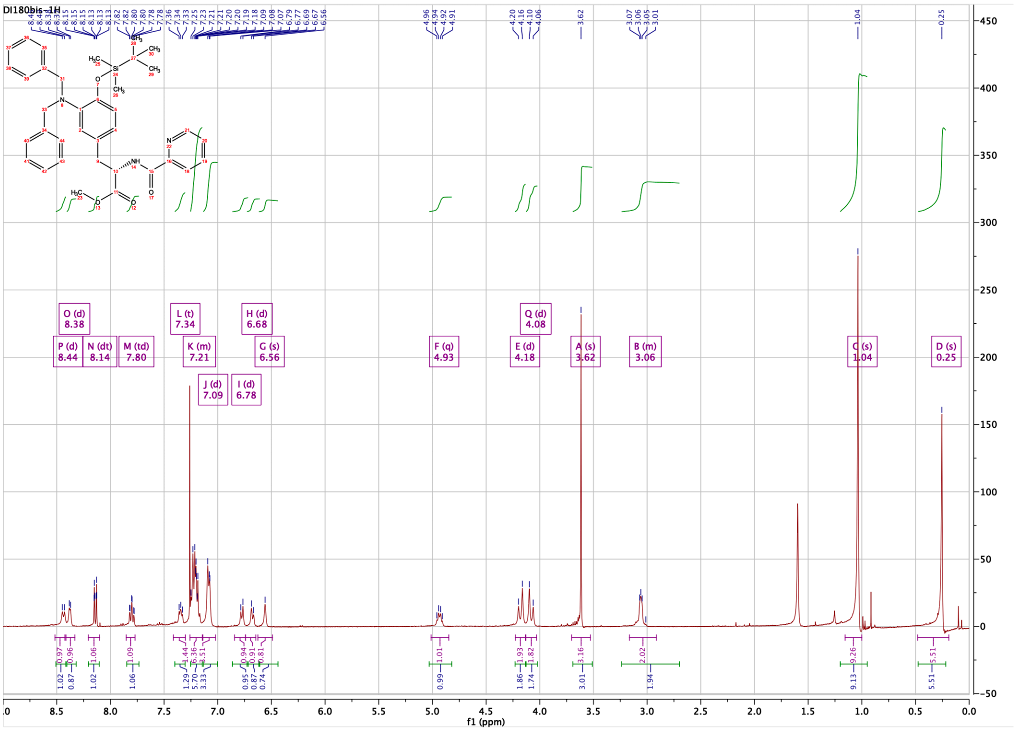

^{1}H NMR (400MHz, $CDCl_3$) compound **5**

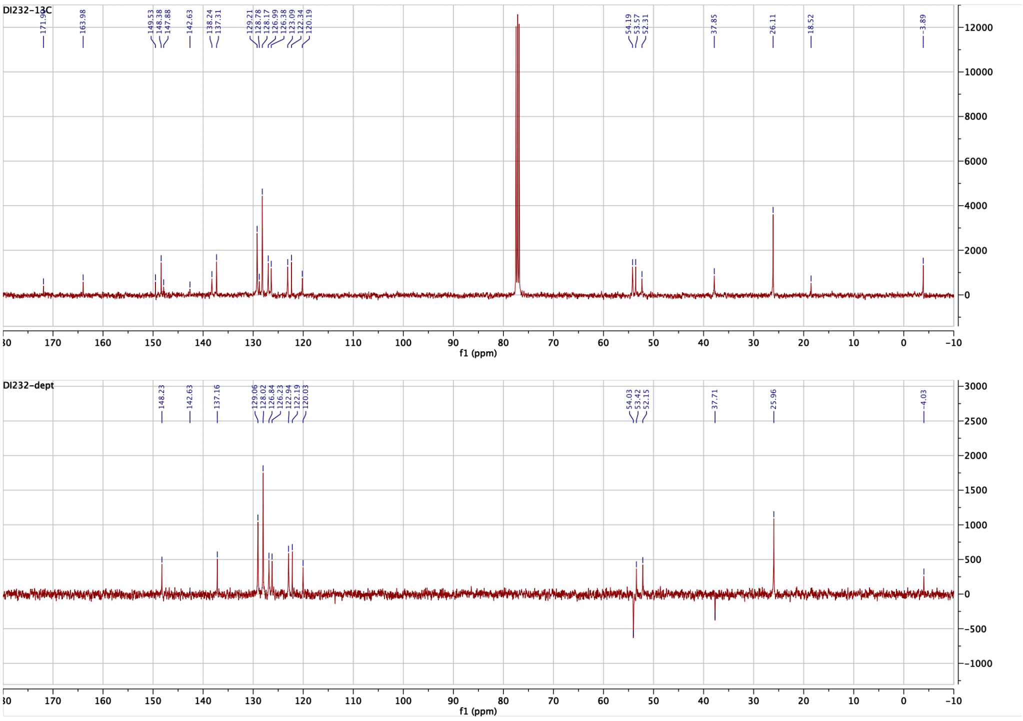

^{13}C and DEPT NMR (101 MHz, $CDCl_3$) compound **5**

Compound 6:

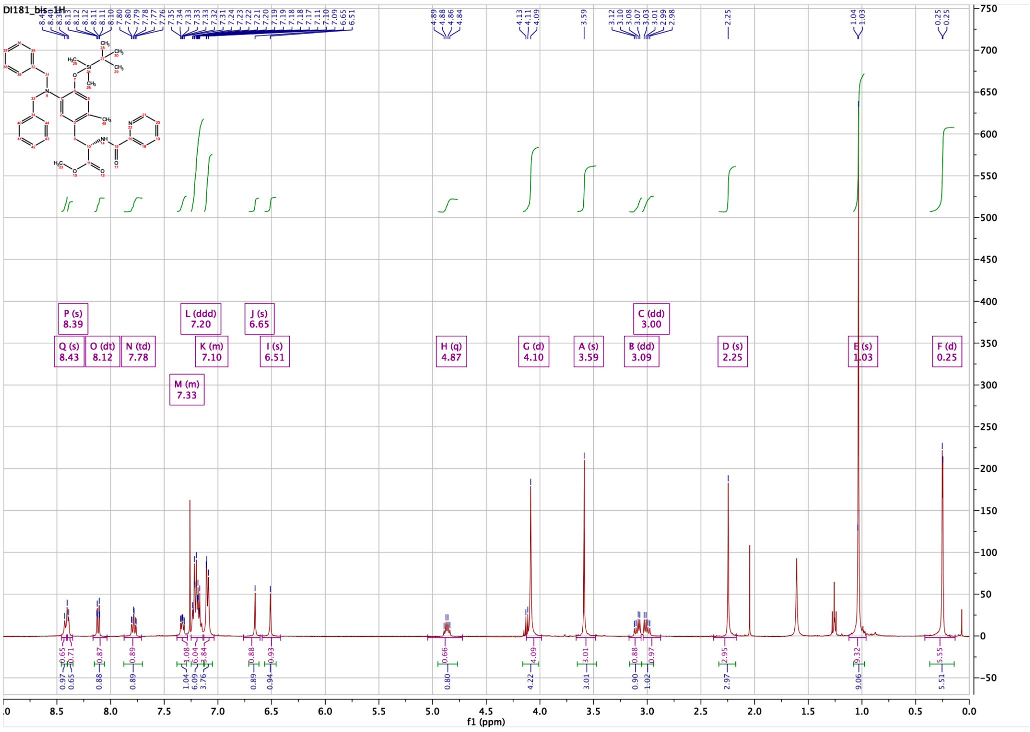

^{1}H NMR (400MHz, $CDCl_3$) compound **6**

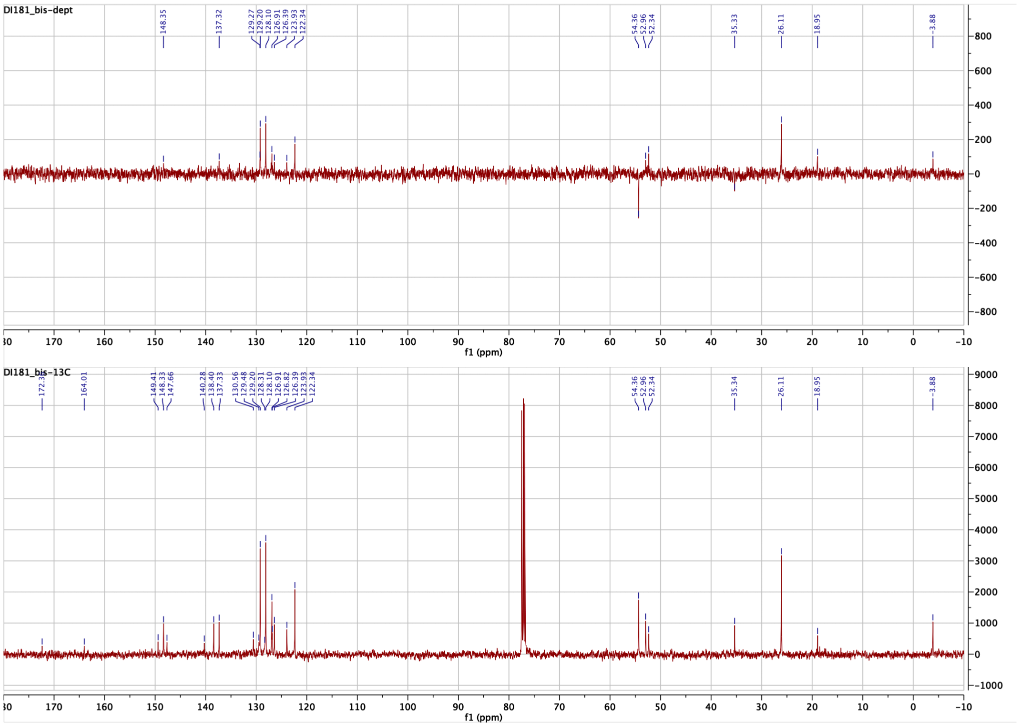

^{13}C and DEPT NMR (101 MHz, $CDCl_3$) compound **6**

Compound 7:

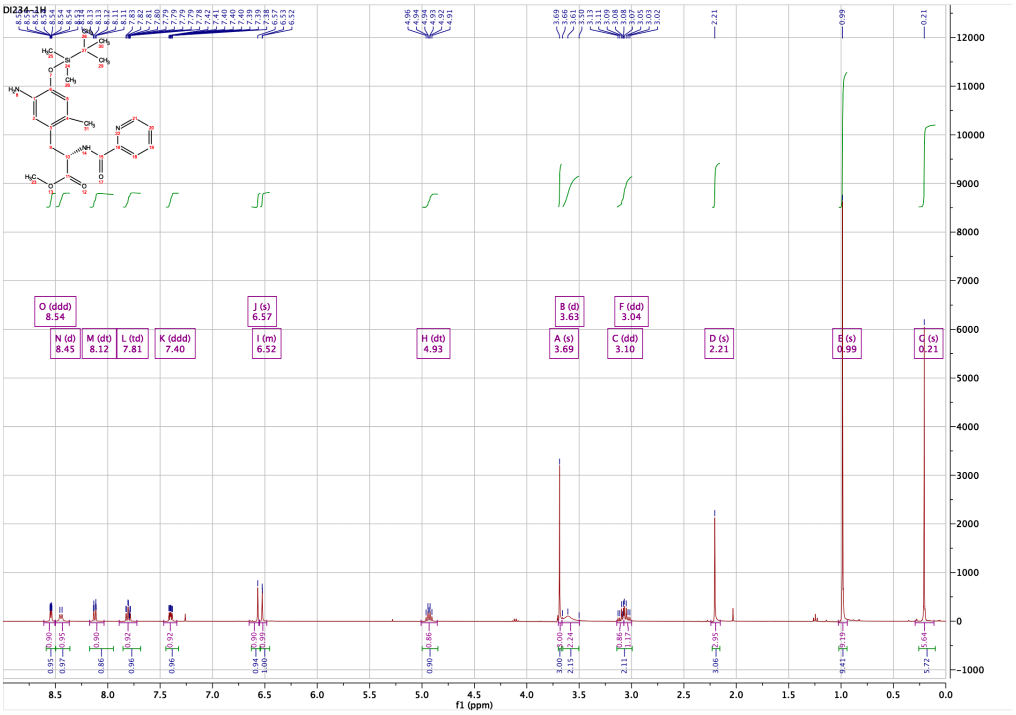

^{1}H NMR (400MHz, $CDCl_3$) compound **7**

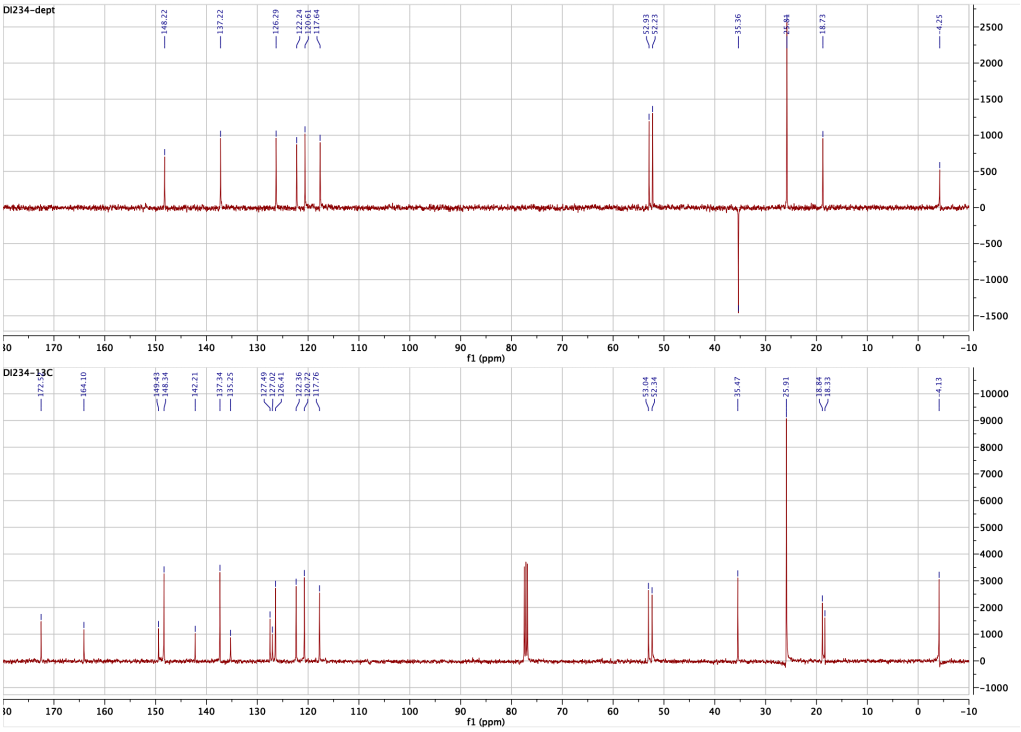

^{13}C and DEPT NMR (101 MHz, $CDCl_3$) compound **7**

Compound 9:

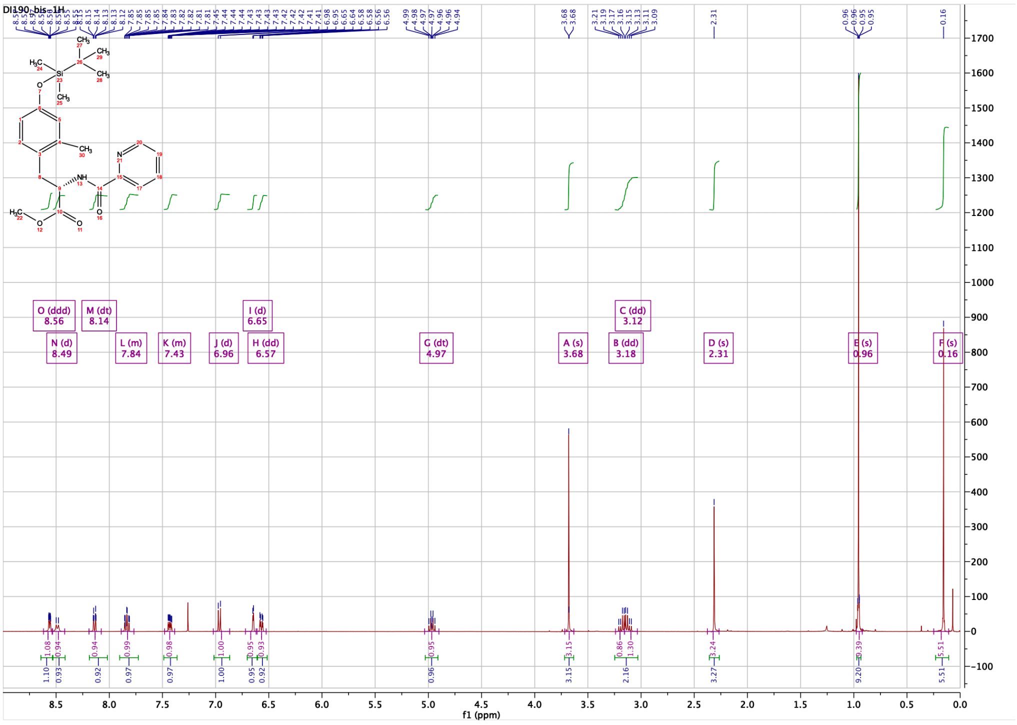

^{1}H NMR (400MHz, $CDCl_3$) compound **9**

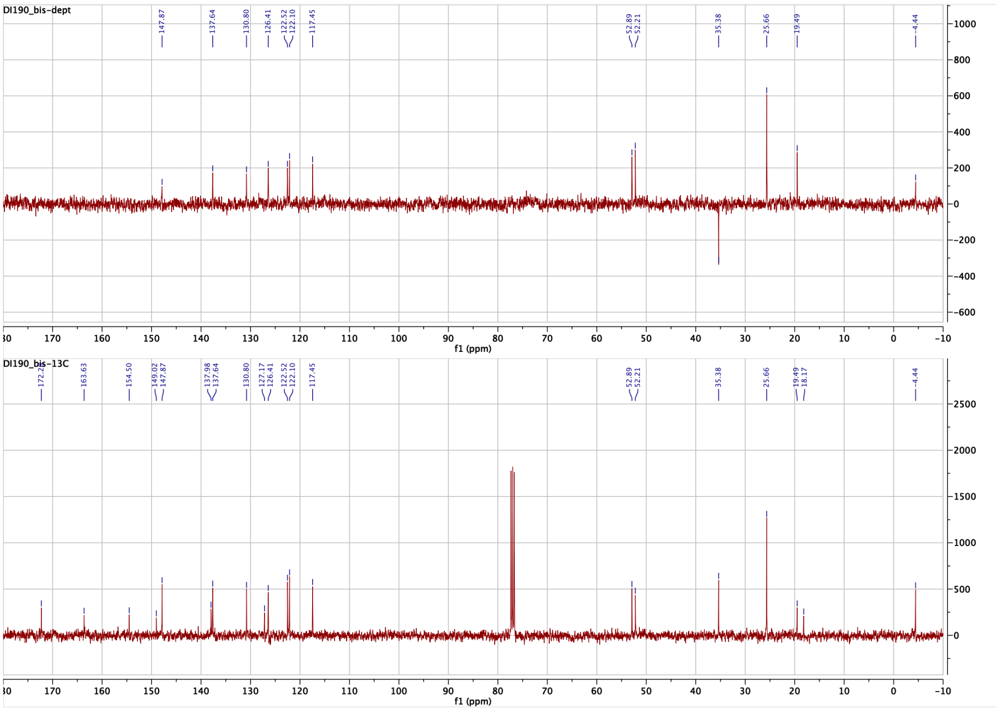

^{13}C and DEPT NMR (101 MHz, $CDCl_3$) compound **9**

Compound 10:

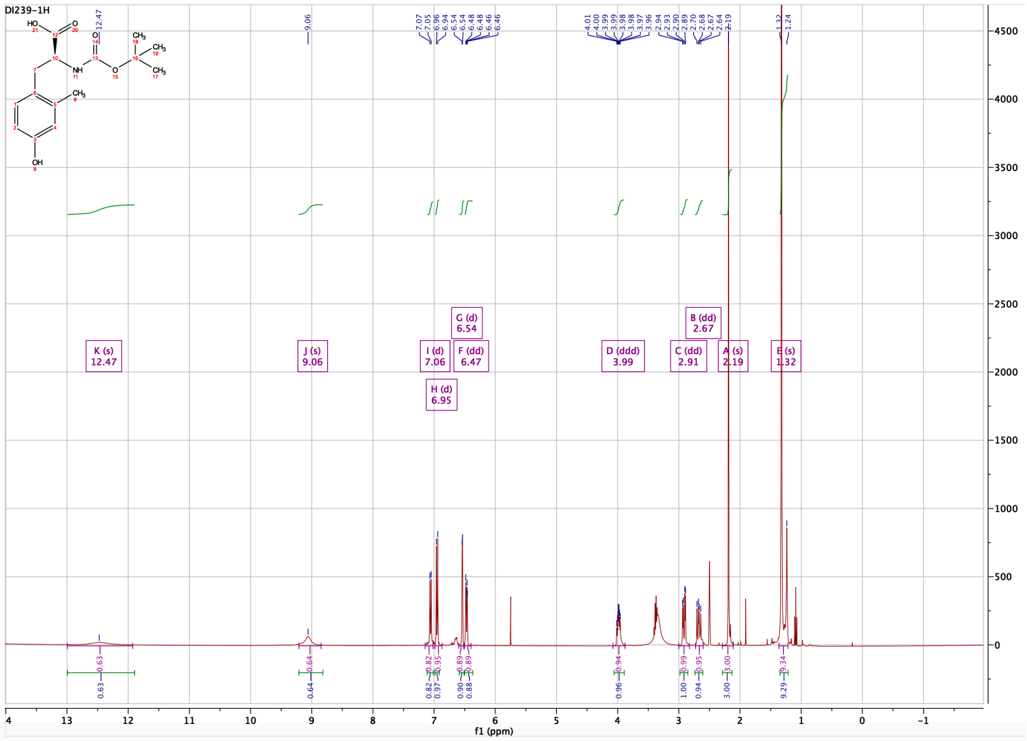

^{1}H NMR (400MHz, DMSO-d$_6$) compound **10**

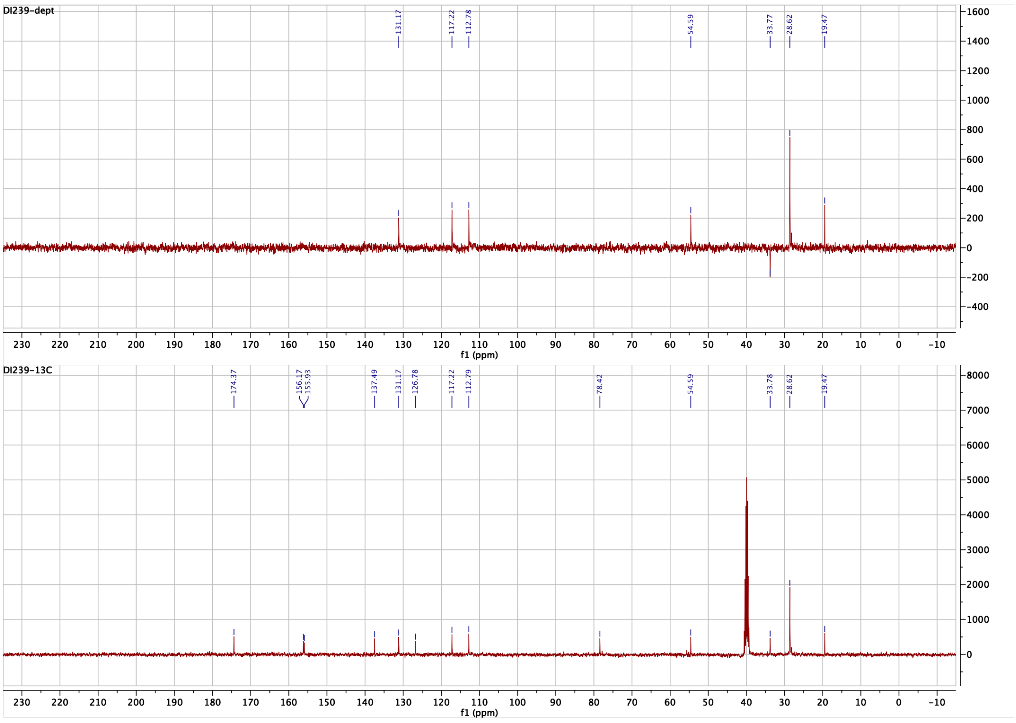

^{13}C and DEPT NMR (101 MHz, DMSO-d$_6$) compound **10**

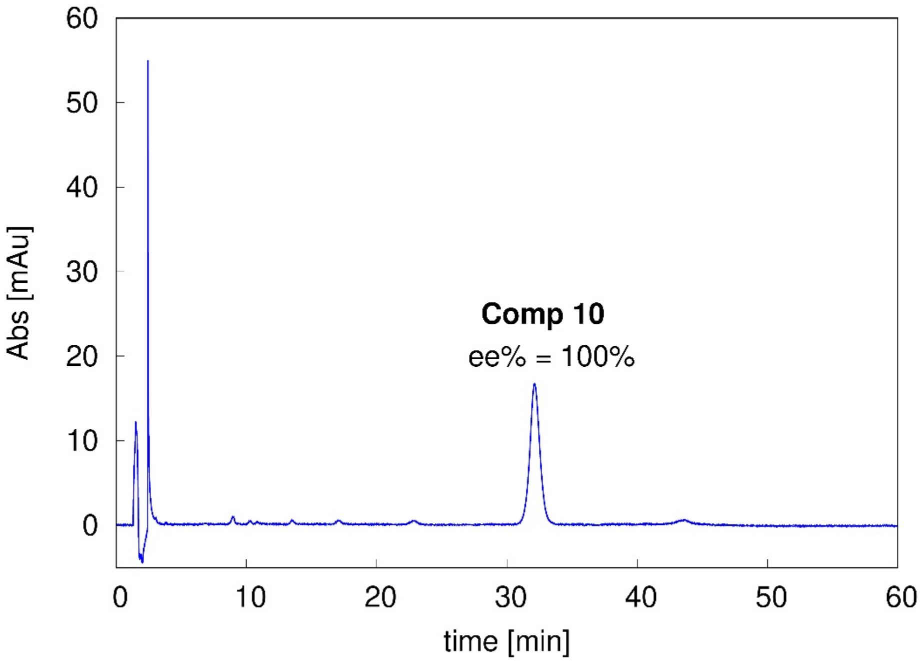

Chromatogram of (L)-Boc-Mmt (compound **10**) (1 mg/mL in methanol) on Whelk01 R,R 150x4.6 mm column packed with 3.5 µm particles size. Isocratic elution at 20% ACN and 80% H_2O (0.1% TFA). Flowrate = 1 mL/min, injection volume = 20 µL, temperature = 25 °C. Retention time = 32.1 min, Area % = 94.8 %, enantiomeric excess = 100%.

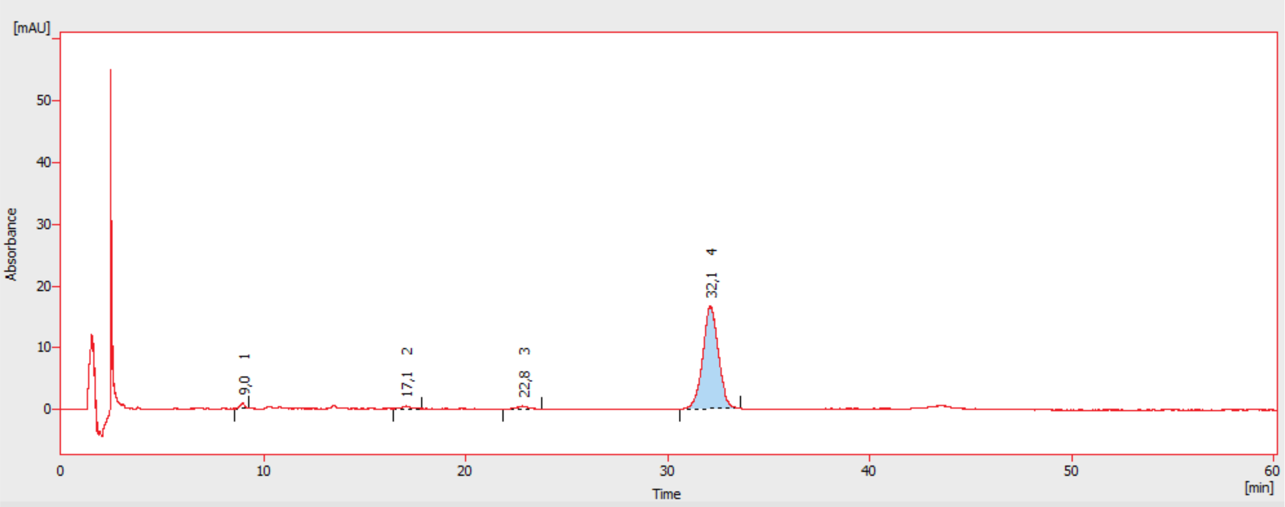

Result Table (Uncal - Data\20231018_122050_whelko1rr_150x4_6_20_boc_mmt_20ul - DAD 2.1L: Channel 1)

	Reten. Time [min]	Area [mAU.s]	Height [mAU]	Area [%]
1	8,985	14,888	0,898	1,6
2	17,062	12,728	0,432	1,4
3	22,843	20,824	0,514	2,2
4	32,113	891,258	16,569	94,8

Chiral HPLC compound **10**

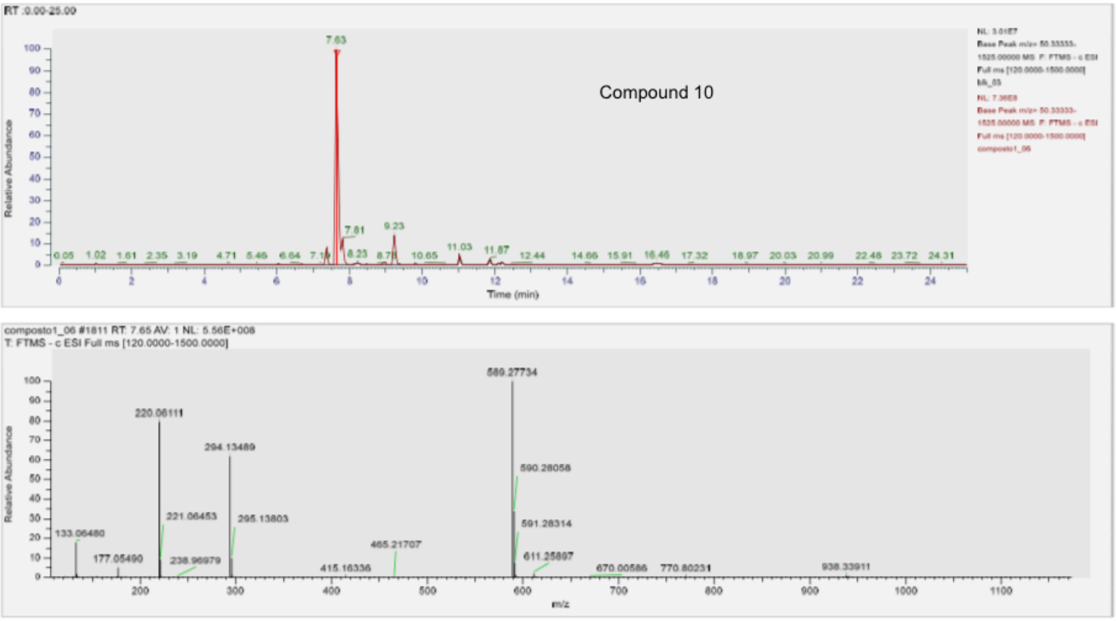

Peak Mass	Display Formula	Combined Fit	RDB	Delta [ppm]	Theo. mass	Rank	Combined Score	# Matched Iso.	# Missed Iso.	MS Cov. [%]	Pattern Cov. [%]	Precursor Ion
294.13489	$C_{15}H_{20}O_5N$	49.6407589	6.5	0.65	294.1347	1	97.35	3	1	100	99.82	[M-H]⁻
589.27734	$C_{30}H_{41}O_{10}N_2$	85.67083182	11.5	1.15	589.27667	1	99.25	5	0	100	100	[2M-H]⁻

HRMS compound **10**

Compound 11:

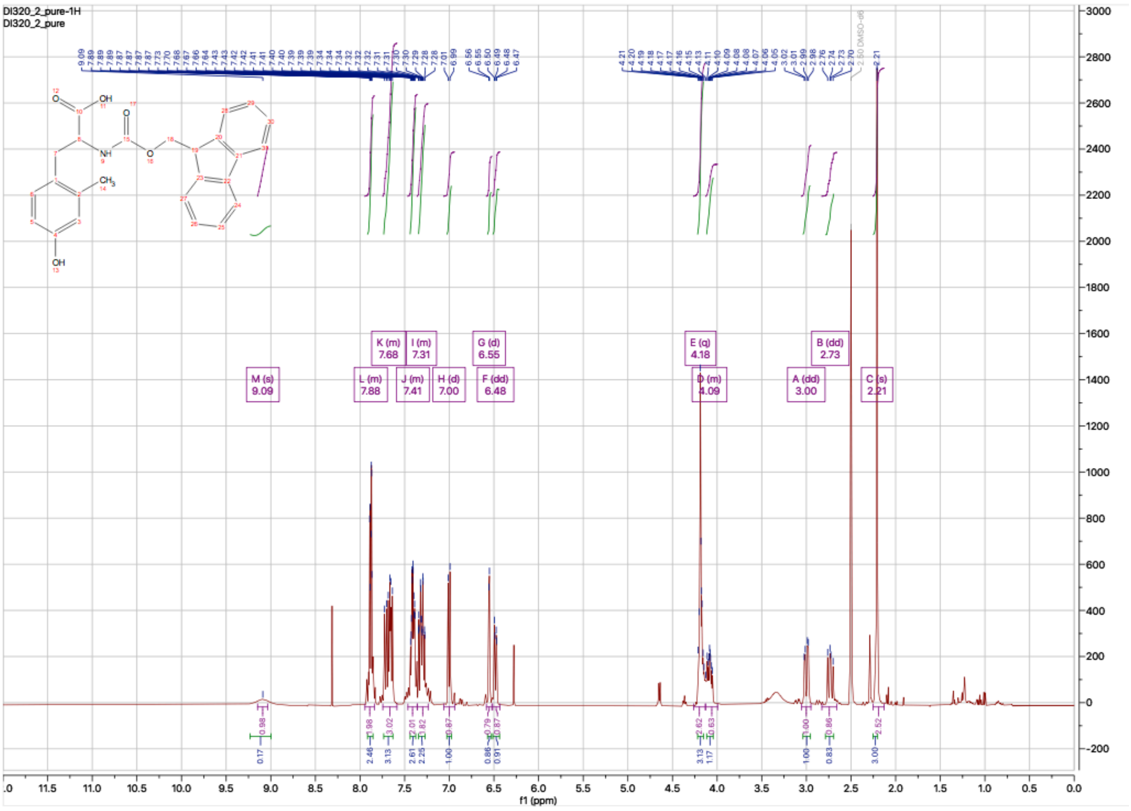

^{1}H NMR (400MHz, DMSO-d_6) compound **11**

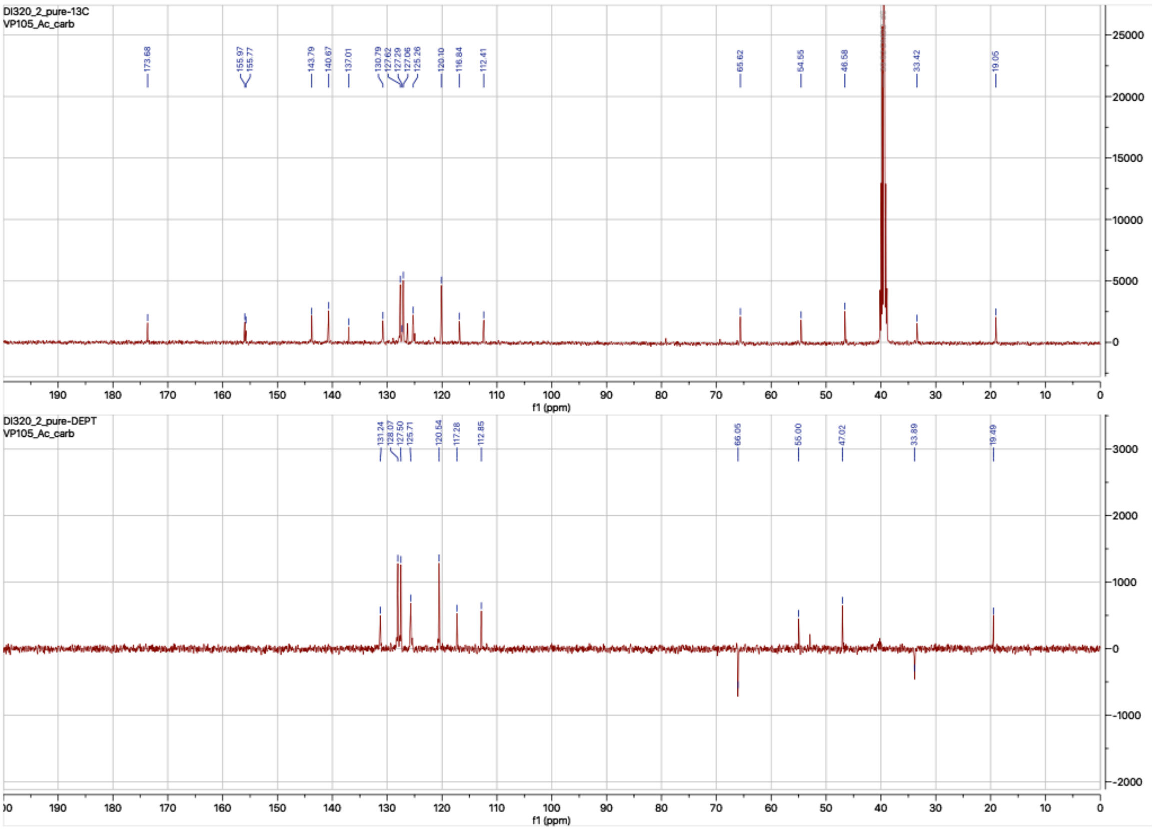

^{13}C and DEPT NMR (101 MHz, DMSO-d_6) compound **11**

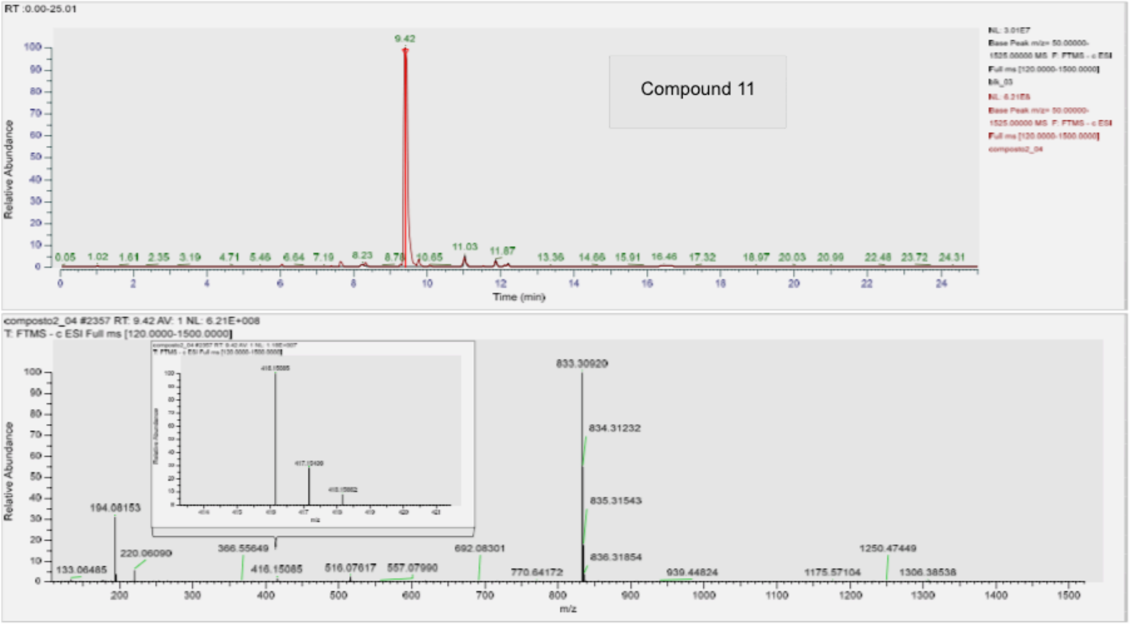

Peak Mass	Display Formula	Combined Fit	RDB	Delta [ppm]	Theo. mass	Rank	Combined Score	# Matched Iso.	# Missed Iso.	MS Cov. [%]	Pattern Cov. [%]	Precursor Ion
833.3092	$C_{50}H_{45}O_{10}N_2$	67.10351336	29.5	1.48	833.30797	3	98.22	5	0	99.95	100	$[2M-H]^-$
416.15085	$C_{25}H_{22}O_5N$	39.71690704	15.5	1.21	416.15035	1	91.04	3	0	93.89	96.6	$[M-H]^-$

HRMS compound **11**

2. Application on SPPS

The compound **11** described was applied in SPPS for the replacement of Phe[1] in the N/OFQ(1-13)-NH_2 peptide sequence. All the peptides were synthesized by taking advantage of the Fmoc/tBu strategy, using an automatic Synthesizer (XP Syro, Biotage Sweden).
Briefly, in SPPS the peptide chain is built up from the *C*-terminal amino acid, anchored to the resin, to the *N*-terminal one, through repeated cycles of alternate *N*-terminal deprotection and coupling reactions with activated *N*-Fmoc-*α*-amino acids. All the amino acids (aa) are orthogonally protected: *N*-Fmoc in the aliphatic α amine and/or Boc, *tert*-butyl (tBu), trityl (Trt), 2,2,5,7,8-pentamethylcromane-6-sulfonyl (Pmc), benzyloxy carbonyl (Z or Cbz) in eventually side chain functional groups. The polystyrene resin Amispheres 20 RAM (20% of its weights is made of polyethylene glicole) was used as starting material. The resin is weighted into an empty syringe, then it is swelled in DMF, at room temperature.
Once the last *α*-amino acid is inserted, the *N*-terminal Fmoc protection is removed, then a single acidic treatment is performed, allowing the removal of side chain protecting groups and the cleavage of the neo-synthesized peptide from the resin. Specifically, the 'cleavage cocktail' used to this aim is composed of trifluoroacetic acid (TFA), water and triethyl silane (Et_3SiH) in 9:0,5:0,5 proportion. The treatment is carried out at room temperature for 3 hours.
The exhausted resin is filtered off, TFA is removed from the filtrate under vacuum, and the neo-synthesized peptide is then precipitated in diethyl ether (Et_2O), isolated through centrifugation and finally purified via reverse phase preparative high-performance liquid chromatography (RP-HPLC) then dried over lyophilization. RP-HPLC analytical gradients were run using a solvent system consisting of A (H_2O + 0.1% TFA) and B (CH_3CN + 0.1% TFA. The conditions used to characterize pure peptides consisted of a linear gradient from 0% to 100% of B solution over 25 min).
The Mmt **11** was incorporated in peptide built by solid-phase peptide synthesis: [(Mmt[1])](N/OFQ(1-13)-NH_2) and it was compared to the [Dmt[1]](N/OFQ(1-13)-NH_2), N/OFQ(1-13)-NH_2 and N/OFQ.

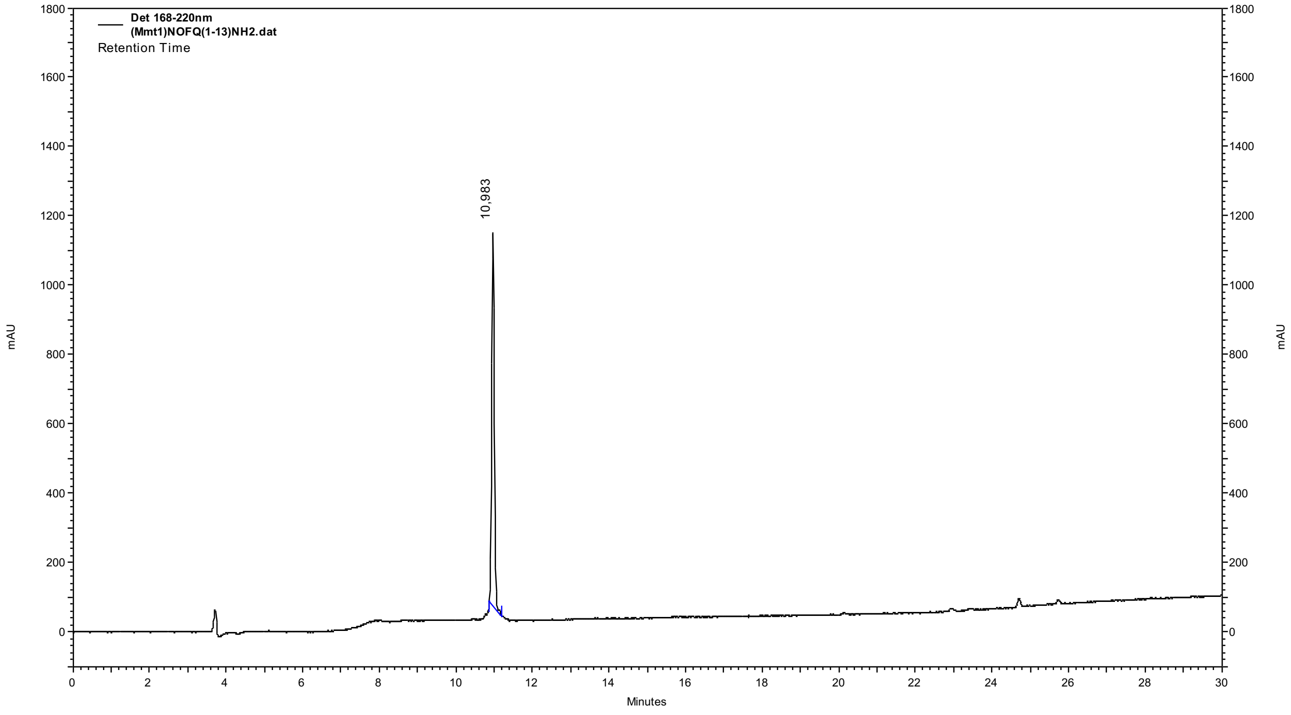

HPLC of compound **11** inserted in position 1 of N/OFQ(1-13)-NH_2

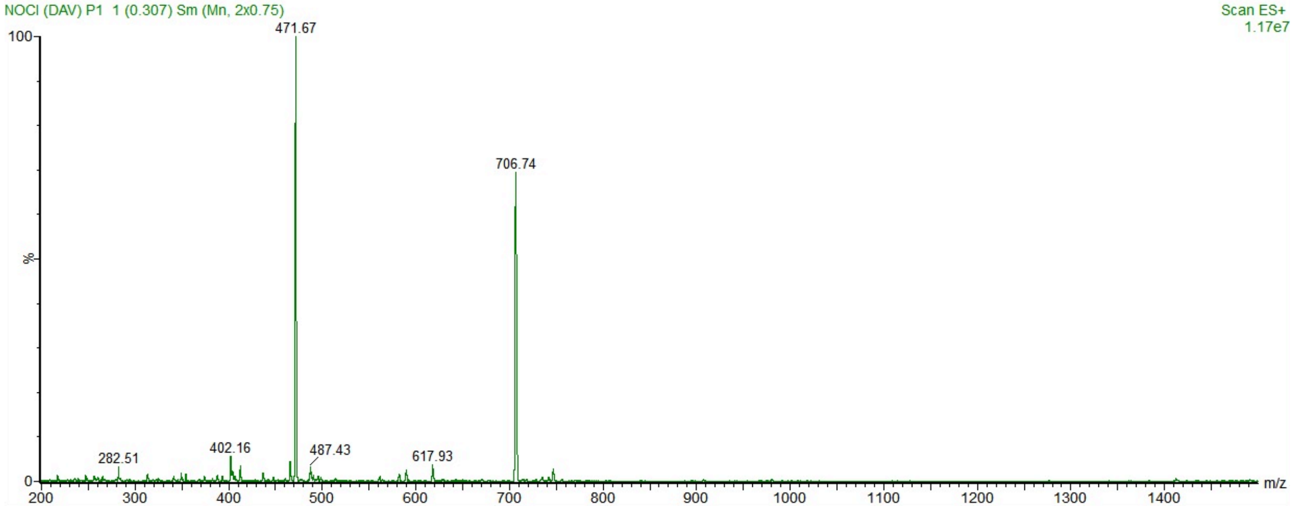

Mass spectrum of compound **11** inserted in position 1 of N/OFQ(1-13)-NH_2

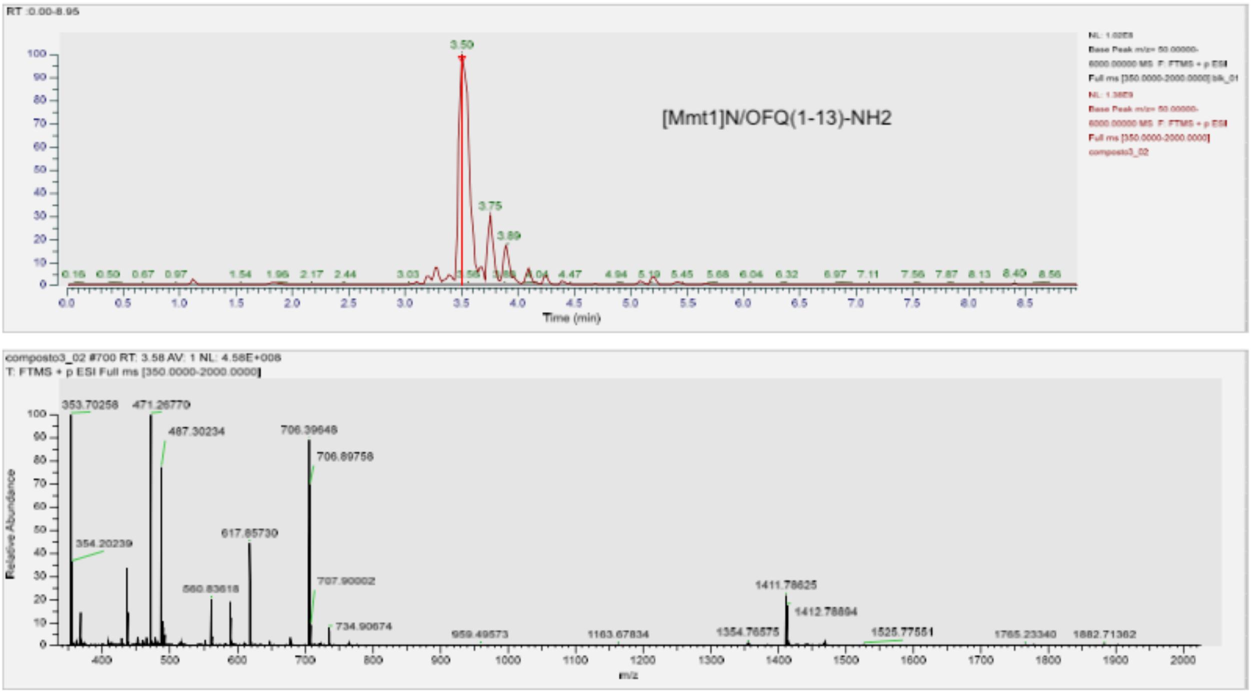

Peak Mass	Display Formula	Combined Fit	RDB	Delta [ppm]	Theo. mass	Rank	Combined Score	# Matched Iso.	# Missed Iso.	MS Cov. [%]	Pattern Cov. [%]	Precursor Ion
1411.78625	$C_{62}H_{103}O_{16}N_{22}$	62.23344667	22.5	-3.85	1411.79169	1	93.76	6	0	95.51	100	$[M+H]^+$
706.39648	$C_{62}H_{104}O_{16}N_{22}$	64.01266112	22	-4.25	706.39948	2	98.11	6	0	100	100	$[M+2H]^+$
471.2677	$C_{62}H_{105}O_{16}N_{22}$	59.58519634	21.5	-2.22	471.26875	1	97.74	6	0	99.86	100	$[M+3H]^+$

HRMS of compound **11** inserted in position 1 of N/OFQ(1-13)-NH_2
